# Monthly Summary

**Published:** 1886-10

**Authors:** 


### Monthly Summary.

---

A PORCELAIN FILLING.—*By J. L. Stokes, D. D. S.*—Some time ago there came a lady into my office who was young and beautiful, but her teeth gave her no little annoyance; some of them were honey-combed on the palatine surface. She called to ask me to fill a depression in the labial surface of a central inscisor; it was a very large cavity through the enamel and even into the dentine. I told her that a gold filling large enough to fill the cavity would be very conspicuous, and advised her to let me insert a filling composed principally of porcelain. She consented, and I proceeded as follows: I took an impression of the labial surface of the tooth with modellng compound, and took a cast from that; then I cut in the tooth on the cast, just such an opening as would have to be made in the natural tooth, then I ground up a piece of porcelain from a porcelain tooth exactly the shade of the natural one. In this way I fastened it to a piece of wood with shellac, so that I could hold it, then I cut it to fit loosely in the cavity in the tooth on cast. I allowed a space to remain between the porcelain and the walls of the tooth all around. I then took the patient in hand again, put on the rubber dam, and cut out just such a cavity in natural tooth as I did in the one on the cast; when I had it thoroughly prepared I filled it with very thin cement (Monogram) and put the porcelain in and pressed it firmly to the bottom of the cavity; this drove out the cement to a great extent, leaving only enough in the cavity to hold the porcelain in place and fill up any irregularities that might have been left in preparing the cement or porcelain. Then I waited until the cement was hard. I took a very fine excavator and removed part of the cement from around the porcelain and started the gold filling. I would fill about one-third of the space at the time, leaving the cement intact to prevent the displacement of the porcelain. I had the cavity counter sunk slightly, and the edge of the porcelain was slightly beveled, which caused the entire space between the walls of the cavity and the porcelain to be counter sunk, when I completed the circular filling of gold around the porcelain. I condensed the gold very carefully, and when it had been completed the gold overlapped the edges of the porcelain very slightly, but firmly, and ran back into a groove cut all around in the opening in the natural tooth sufficiently to hold the gold itself in place. When it was polished it looked like a thread of gold, or a very small gold ring let into the tooth.

This operation made the already beautiful and attractive young lady even more so.

Don't it make us happy when we do a beautiful piece of work? and the more difficult it is the happier we are.

I'd give this advice to the younger members of the profession, (the older heads would think me "saucy") always try and improve on every operation you do, and if you have a set of teeth in your charge, in which there is already beautiful work, try and improve on it. This is our duty.—*Southern Dental Journal.*

---

WIRE LIGATURES.—*By Geo. S. Staples, Sherman, Texas.*—DR. CATCHING: As I promised to let you hear from me occasionally, I will begin with an article on the use of wire ligatures. I had hoped to have demonstrated their use at Nashville, but not having had an opportunity to do so, I give it to the profession through your valuable journal, (provided you think it worth publishing.) I have tried almost all kinds of clamps for the filling of buccal and labial cavities and have never yet found *one* to give satisfaction. So a few months since I had a very difficult cavity on the buccal wall of second left sup. bicuspid. As I anticipated a great deal of trouble with it, I commenced to study some plan by which I could overcome the difficulty, when I concluded to try common binding wire, which I did with the happiest results. Since the first trial I have relegated all clamps for such purposes to the dogs ("or any other power") and now such cavities have no terrors for me any more. In fact, I have not only found wire ligatures the thing for buccal and labial cavities, but on all cavities that extend under the gums, when not so far back as to be unable to apply it.

Now if you think this worth anything you can publish it, otherwise stick it in the waste basket, and there will be no harm done. Any how try the wire yourself and see how you like it.—*Southern Dental Journal.*

